# Antibiotic use in township hospitals during the COVID-19 pandemic in Shandong, China

**DOI:** 10.1186/s13756-022-01206-8

**Published:** 2022-12-24

**Authors:** Ting Wang, Liyan Shen, Jia Yin, Liansheng Zhou, Qiang Sun

**Affiliations:** 1 Center for Health Management and Policy Research, School of Public Health, Cheeloo College of Medicine, Shandong University, Jinan, Shandong Province China; 2 National Health Commission (NHC) Key Lab of Health Economics and Policy Research, Shandong University, Jinan, Shandong Province China; 3 Zhucheng Center for Disease Control and Prevention, Zhucheng, Shandong Province China; 4 Shandong University, Baotuquan Campus, #44 Wenhua Xi Road, Jinan, 250012 Shandong Province China

**Keywords:** Antibiotic consumption, COVID-19, Primary healthcare settings, China

## Abstract

**Background:**

The overuse of antibiotics in primary healthcare settings (PHSs) has caused a serious public health problem in China. The outbreak of the Coronavirus Disease-19 (COVID-19) pandemic brought about dramatic changes in the supply of and demand for medical services in PHSs, possibly resulting in unprecedented changes in antibiotic use.

**Objective:**

This study aims to assess the immediate and long-term impacts of the COVID-19 pandemic on the changes in antibiotic consumption in PHSs.

**Method:**

The data on antibiotic consumption were collected from selected township hospitals in Shandong, China from January 2019 to December 2021. Antibiotic consumption was quantified by using the defined daily doses (DDDs) and the WHO Access, Watch, Reserve category. A segmented regression model was established to analyze the immediate and long-term impacts of the COVID-19 pandemic on antibiotic use by using the interrupted time series analysis.

**Results:**

The overall antibiotic consumption in all PHSs decreased by 32.04% and 16.69% in 2020 and 2021 respectively compared to the corresponding period in 2019. Over the entire study period, the use of penicillins (J01C) and cephalosporins (J01D) accounted for more than 50% of the total antibiotic consumption. The average annual consumption of Watch category antibiotics decreased by 42.02% and 33.47% in 2020 and 2021 respectively compared to that in 2019. According to the interrupted time series analysis, the total antibiotic consumption decreased significantly immediately after the COVID-19 pandemic outbreak (coef. = − 2.712, *p* = 0.045), but it then increased significantly over a long-term (coef. = 0.205, *p* = 0.005). Additionally, the consumption of Access category antibiotics increased significantly in PHSs in the long-term (coef. = 0.136, *p* = 0.018). However, the consumption of Watch category antibiotics declined sharply immediately after the pandemic (coef. = − 1.222, *p* < 0.001), but then it increased slightly over a long-term (coef. = 0.073, *p* < 0.001).

**Conclusion:**

The extensive use of penicillin and cephalosporins should be of great concern. After the outbreak of COVID-19 pandemic, the total antibiotic consumption decreased generally and the use pattern was improved to some extent in the PHSs in Shandong, China. This provides an opportunity for improving the misuse of antibiotics in PHSs in China.

**Supplementary Information:**

The online version contains supplementary material available at 10.1186/s13756-022-01206-8.

## Introduction

Antimicrobial resistance (AMR) is a major urgent threat to global public health, and it has imposed a huge medical and economic burden on the whole society [1–3]. According to the report of the World Health Organization (WHO), antibiotic resistance has given rise to a global public health crisis, considering the fact that the public health is threatened, the use of antibiotics has to be optimized [4].

As one of the largest consumers of antibiotics, China plays an important role in containing and mitigating AMR [5]. Antibiotics are the most commonly prescribed drugs in PHSs [6, 7]. In China, more than 50% of outpatients have been prescribed antibiotics over the past decade [8], far above the level recommended by WHO (less than 30%) [9]. Therefore, it is necessary to optimize antibiotic use in PHSs for containing bacterial resistance [4, 6, 10].

The Coronavirus Disease-19 (COVID-19) pandemic swept across the world quickly and placed a heavy burden on the health systems globally. The impacts of the COVID-19 pandemic on antibiotic consumption and the risk of AMR have become a concern of many researchers of antibiotic resistance [11–19]. According to a report, after the outbreak of the COVID-19 pandemic in most European countries, the total consumption of antibiotics declined by more than 15%, which was mainly observed in PHSs [13]. Several national studies conducted in the USA, Portugal, Canada, and the UK also showed that the overall antibiotic consumption by outpatients or broad-spectrum antibiotic prescriptions decreased significantly during the COVID-19 pandemic. Especially, the number of prescriptions of certain categories of antibacterials (e.g., third-generation cephalosporins, fluoroquinolones) decreased significantly [11, 12, 14–16]. Similarly, several studies in China also found that there was a decline antibiotic consumption during the COVID-19 pandemic [17–19]. For example, a study from Yinchuan of China showed that the decrease in inappropriate antibiotic prescriptions in primary care settings may be associated with the COVID-19 pandemic [19]. Therefore, the antibiotic use pattern may have changed greatly during the COVID-19 pandemic. However, no adequate evidence has been found for the changes in antibiotic consumption in PHSs in China.

This study aimed to assess the immediate and long-term impacts of the COVID-19 pandemic on the changes in antibiotic consumption in PHSs in Shandong, China. The findings of this study may be conducive to developing and implementing intervention and management policies for antibiotic consumption in PHSs to further improve the misuse of antibiotics.

## Methods

### Study setting and design

This study was conducted in Shandong province in eastern China, which had a total population of 101, 699, 900 at the end of 2021 [20]. The method of purpose sampling was used in the study. Two counties S and Y in City L located in the western area of Shandong Province were selected as the target locations. The two counties have similar mid-level development indexes in terms of Gross Domestic Product (GDP), number of permanent residents, number of health technicians, and number of township hospitals. In addition, the pandemic prevention and control policies were highly synchronized during the COVID-19 pandemic in PHSs in Shandong, China. Therefore, the sample township hospitals are representative of the majority of township hospitals in rural Shandong. They can representatively display the changes of antibiotic consumption trend and the use pattern before and after the outbreak of the COVID-19 pandemic.

Township hospitals are primary healthcare centers in rural China. Their primary missions are to provide rural population with extensive primary outpatient services (i.e., establishment resident health records, common diseases treatment, non-communicable diseases control, vaccination and health education) and limited inpatient service (i.e., basic clinical examination, surgical treatment for a few diseases). Antibiotics are used by inpatients and outpatients, but most of them are consumed by outpatients of the township hospitals in Shandong, China. During the early period of the COVID-19 pandemic, the township hospitals changed their daily work and business hours. For example, they greatly shortened their opening hours, and most of the medical staff were deployed to assist in tracking down infected individuals and conduct epidemiological investigation. At the same time, the number of patients in township hospitals decreased sharply, for those, patients with fever were transferred to COVID-19 pandemic specific clinics. In addition, antibiotics in pharmacies were not available for patients without physicians’ prescription. Instead, antibiotics were mostly prescribed in PHSs in rural China.

Data were collected from all township hospitals (38 in total) in counties S and Y in City L in Shandong Province. In Shandong Province, PHSs are only allowed to purchase, store and distribute drugs listed in the *Regulations on Drug Administration* based on the zero price difference policy. At the same time, doctors in PHSs are only permitted to prescribe antibiotics listed in the *List of Essential Drugs* [21].

We conducted a 3-year natural, before and after, quasi-experimental study in the real world. A controlled interruption time series design was used to collect data at several time points before and after the COVID-19 pandemic outbreak, aiming to investigate the immediate and long-term impacts of the pandemic on antibiotic consumption. In this study, the effectiveness of data was estimated by controlling the baseline level and the trend [22], the longitudinal characteristics of the data ensured the robustness of results [23].

### Data collection and management

We retrospectively collected aggregated monthly antibiotic consumption data from 38 township hospitals in counties S and Y of City L in Shandong, China, from January 2019 to December 2021. The data were collected with the following method. Two researchers were responsible for preparing Excel forms with headers. These persons emailed feedback to the researchers. At the end of December 2019, and then it spread to Shandong Province due to population migration during Chinese New Year holidays [24]. Therefore, the antibiotic consumption data over 12 months before and 24 months after the outbreak of the COVID-19 pandemic was included. The antibiotic consumption data mainly included the following variables: names of township hospitals, manufacturer of antibiotics, unique chemical substance name, generic name, dosage form, unit strength, specification, unit (by box, bottle, or ampoule), price per unit, inventory at the beginning of the month, monthly purchase quantity, monthly ex-warehouse quantity, monthly inventory at the end of the month, etc.

The original data were managed in Microsoft Excel (version 2019). We strictly controlled the quality of the data, carefully sorted and summarized them after receiving the emails from the persons in charge of the township hospitals. The validity of the data was assessed by two researchers engaged in antibiotic resistance research. They mainly made logic proofreading and supplemented outliers or missing values. During the process of cleaning data, the data with problems were separately listed and sent back to the corresponding persons in charge via emails for supplementing or correction. For example, they should supplement monthly usage, specifications, and dosage forms of drug, and check whether the end-of-month inventory of the drugs as consistent with the beginning-of-month inventory of the next month.

We assessed antibiotic consumption data according to Anatomical Therapeutic Chemical (ATC) classification J01 (i.e., antibacterial for systemic use) [25]. Additional file 1: Table S1 provides a full list of antibiotics analyzed in this study. A total of 55 unique chemical substance names (55 ATC-5 codes) were identified for single or combined antibiotics; they were first classified into 19 ATC-4 classes and secondly into 6 ATC-3 groups. At the same time, antibiotics were classified according to the WHO AWaRe categories (version 2021), aiming to analyze the use patterns of antibiotics [26]. Only one type of antibiotic, Fosfomycin, was classified as Reserve. Therefore, the antibiotics of the Reserve category were not included in the analysis. Instead, only Access and Watch category antibiotics were included. The Access category antibiotics are recommended by the WHO as the first or second choice for empirical treatment, whereas the increased consumption of Watch category antibiotics will aggravate antibiotic resistance.

### Outcome measures

The study assessed the immediate and long-term impacts of the COVID-19 pandemic on the changes in antibiotic use. The primary outcome indicator of interest was the monthly antibiotic consumption. Antibiotic consumption was measures using Defined Daily Dose (DDD), an assumed average maintenance dose developed by WHO to compare drug consumptions [25]. In this study, the DDD value of each drug was determined according to the Guidelines for ATC classification and DDD assignment 2021 [26]. The DDD equivalence per package [DPP = (unit strength * package size/DDD)] of drugs was calculated according to ATC templates. The total consumption of each group of procured drugs (DDDs) was estimated as the summed DPPs of all-inclusive products [27].

$$DDD_{s} = \mathop \sum \limits_{i = 1}^{n} \left( {DPP_{i} \times N_{i} } \right)$$

where *N*_*i*_ represents the number of packages of a certain antibiotic product(*i*) used in the township hospitals.

### Data analysis

This study used the descriptive statistical method to quantify the patterns and trends of antibiotic consumption. First, the relative changes in the overall antibiotic consumption among all PHSs in 2020 and 2021 compared to the corresponding periods in 2019 were described. Second, a monthly antibiotic consumption trend chart based on ATC classification and WHO AWaRe category was prepared to observe and describe changes in antibiotic consumption from January 2019 to December 2021, respectively.

By using interrupted time series analysis, the immediate and long-term impacts of the COVID-19 pandemic on the trends of antibiotic consumption in PHSs were assessed [23, 28]. The time unit in this study was month, for all the data were collected monthly at even intervals. With December 2019 taken as the intervention time point, the data over 12 months before the intervention and 24 months after the intervention was finally included in the study. A segmented regression model was established as follows:

$$Y_{t} = \beta_{0} + \beta_{1} \times Time_{t} + \beta_{2} \times Covid{ - 19}_{t} + \beta_{3} \times Long{ - }term_{t} + \beta_{4} \times Cold + \varepsilon_{t}$$

where $Y_{t}$ is the independent outcome variable of month t (antibiotic consumption DDDs); where $Time_{t}$ is a continuous time series variable (1,2,3… 36); *Covid-19*_*t*_ represents the intervention time indicator before and after the outbreak of the COVID-19 pandemic. The intervention time nodes lie in the 12th month before the outbreak of COVID-19 pandemic (t = 0) and the 24th after the outbreak of COVID-19 pandemic (t = 1); where *Long-term*_*t*_ represents the time since the outbreak of the COVID-19 pandemic; its value before the outbreak of COVID-19 pandemic is 0, while its value after the outbreak of COVID-19 pandemic is 1, 2, 3, …24, corresponding to January 2020 to December 2021 (long-term effect). In addition, a dummy variable *Cold *was set to control the extreme value of antibiotic consumption during the coldest period of Chinese Spring Festival, which was the wild data point of this study [28–31]. By referring to previous studies, we assigned 1 to the variable *Cold* in December and January of each year, and 0 for the rest of months of these the year [32].

In this model, *β*_*0*_ is used to estimate the level of antibiotic consumption at the beginning of the time series. *β*_*1*_ is used to estimate the change trends of antibiotic consumption prior to the outbreak of the COVID-19 pandemic. *β*_*2*_ is used to assess the changes immediately after the outbreak of the COVID-19 pandemic. *β*_*3*_ reflects the monthly changes in antibiotic consumption after the outbreak of the COVID-19 pandemic. If it is different from the trend before the outbreak of the COVID-19 pandemic, it suggests that the outbreak of the COVID-19 pandemic has a long-lasting impact on the antibiotic consumption. *β*_*4*_ is used to estimate the weather effect of the coldest period. $\varepsilon_{t}$ is an estimate of the random error at time t. In addition, Durbin–Watson test was performed to verify the existence of the first-order autocorrelation (if the value is about 2, it indicates that there is no autocorrelation) [33]. In case of an autocorrelation, the regression will be estimated using the Prais-Winsten method [32].

All statistical analyses were performed in STATA version 15.0 (STATA Crop LP, College Station, TX, USA), and *P* < 0.05 was considered statistically significant.

## Results

### Overall antibiotic consumption

Generally, the antibiotic consumption decreased by 32.04% and 16.69% from January to December in 2020 and 2021 compared to the corresponding periods in 2019, respectively (Table 1).

**Table 1:** Monthly comparative analysis of overall antibiotic consumption in PHSs

	Before	During the COVID-19 pandemic		% Relative change (2020 vs 2019)	%Relative change (2021 vs 2019)
	2019	2020	2021		
*Antibiotic consumption (ten thousand DDDs)*					
Total	170.36	115.78	141.93	− 32.04	− 16.69
January	19.14	12.47	12.95	− 34.85	− 32.34
February	16.71	9.69	10.56	− 42.01	− 36.80
March	14.81	9.69	11.48	− 34.57	− 22.48
April	13.64	8.43	10.17	− 38.20	− 25.44
May	13.91	8.43	10.17	− 39.40	− 26.89
June	12.15	8.12	10.59	− 33.17	− 12.84
July	12.37	8.58	11.31	− 30.64	− 8.57
August	13.07	8.92	12.99	− 31.75	− 0.61
September	12.29	9.86	8.81	− 19.77	− 28.32
October	13.04	9.86	13.07	− 24.39	0.23
November	13.35	9.59	14.49	− 28.16	8.54
December	15.88	12.14	15.34	− 23.55	− 3.40

*DDDs* defined daily doses

### Antibiotic consumption by ATC classification

In this study, the categories of the most frequently used antibiotics were J01C (beta-lactam antibacterials, penicillins), followed by J01D (cephalosporins, other beta-lactam antibacterials), J01F (macrolides, lincosamides and streptogramins) and J01M (quinolone antibacterials).

**Fig. 1:**
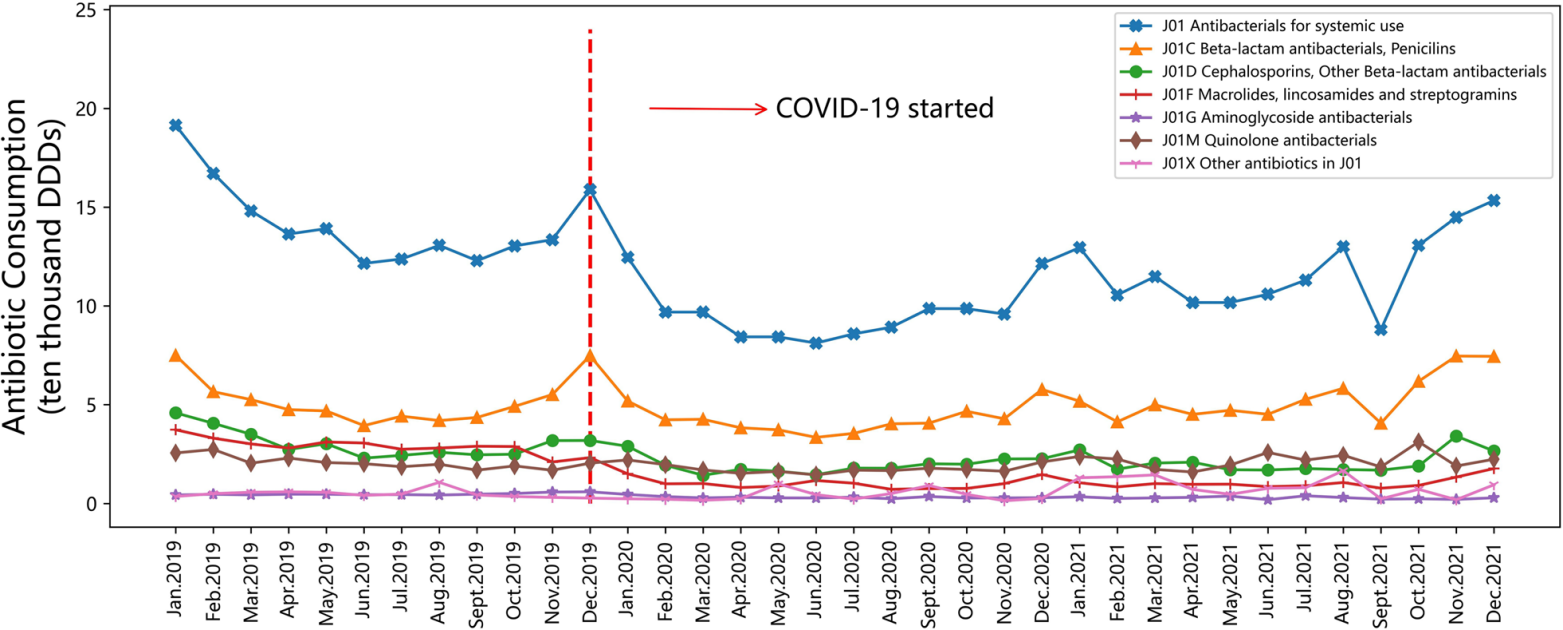
Monthly changes in antibiotic consumption by ATC classification

According to Fig. 1, the consumption of J01C antibiotics (beta-lactam antibacterial, penicillins) fluctuated throughout the study period. It declined sharply in December 2019. Then, it fluctuated steadily on a relatively low level. However, it increased significantly in September 2021. This trend mainly affected the change of the total consumption of J01 antibiotics during this period.

### Antibiotic consumption by the WHO AWaRe category

**Fig. 2:**
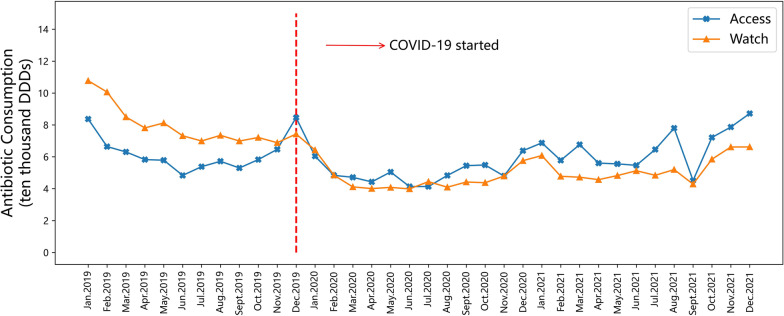
Monthly changes in antibiotic consumption by WHO AWaRe category

According to Table 2, the average annual consumption of Watch category antibiotics decreased by 42.02% and 33.47% in 2020 and 2021 compared to the same periods in 2019, respectively. Figure 2 illustrates that before the outbreak of COVID-19, Watch category antibiotics were consumed much more than Access category antibiotics. However, in December 2019 (after the outbreak of COVID-19), the trend of antibiotic consumption was reversed. It suggests that the outbreak of the COVID-19 pandemic may have improves the improper antibiotic consumption patterns in PHSs.

**Table 2:** Annually comparative analysis of antibiotic consumption by WHO AWaRe category in PHSs

AWaRe category	Before	During the COVID-19 pandemic		% Relative change (2020 vs 2019)	%Relative change (2021 vs 2019)
	2019	2020	2021		
Access antibiotics (ten thousand DDDs)	74.91	60.27	78.62	− 19.54	4.95
Watch antibiotics (ten thousand DDDs)	95.44	55.34	63.50	− 42.02	− 33.47

*DDDs* defined daily doses

### Impacts of the COVID-19 pandemic on antibiotic consumption

**Fig. 3:**
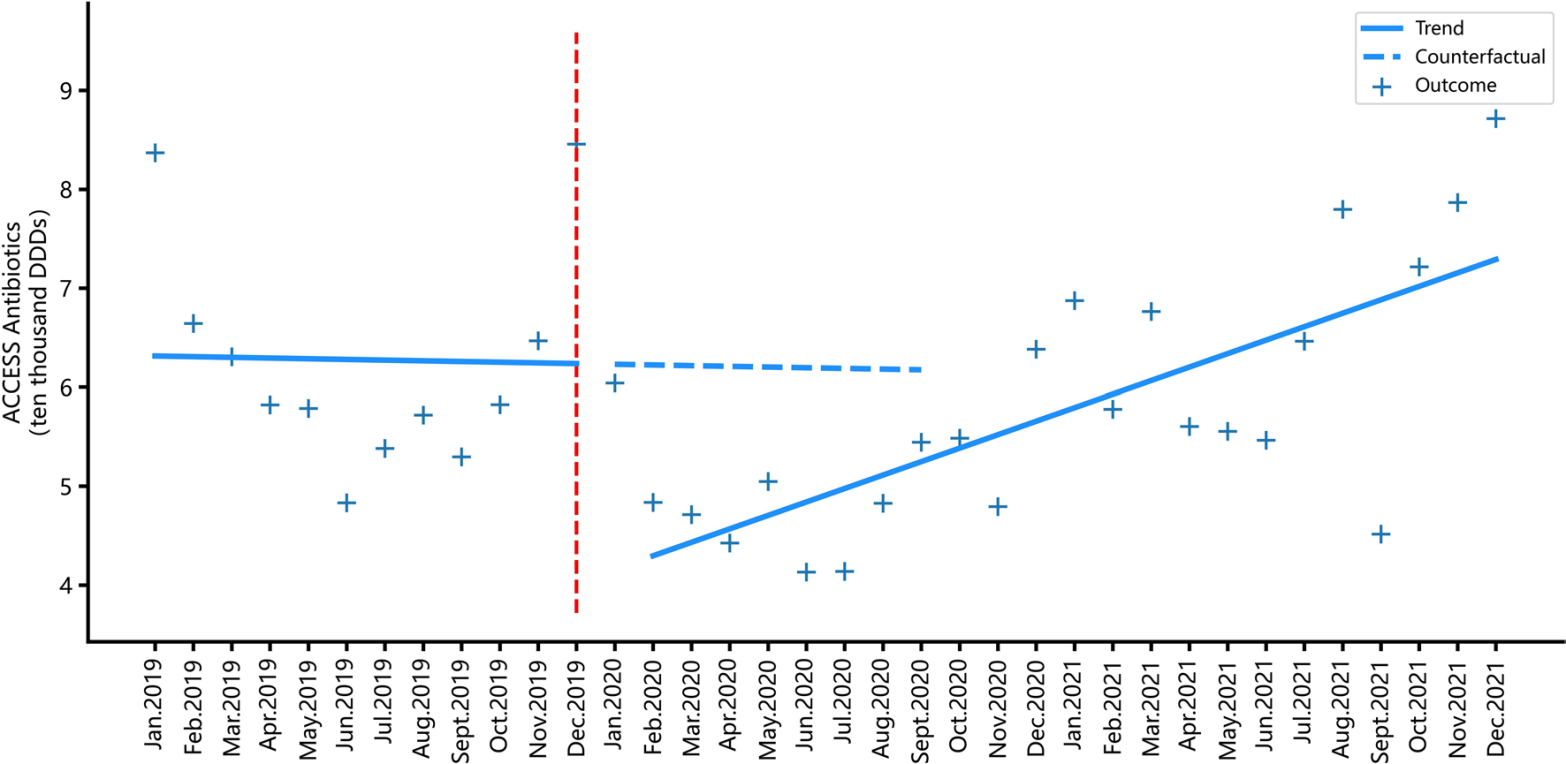
Monthly consumption trend of antibiotics from the Access category in PHSs

**Fig. 4:**
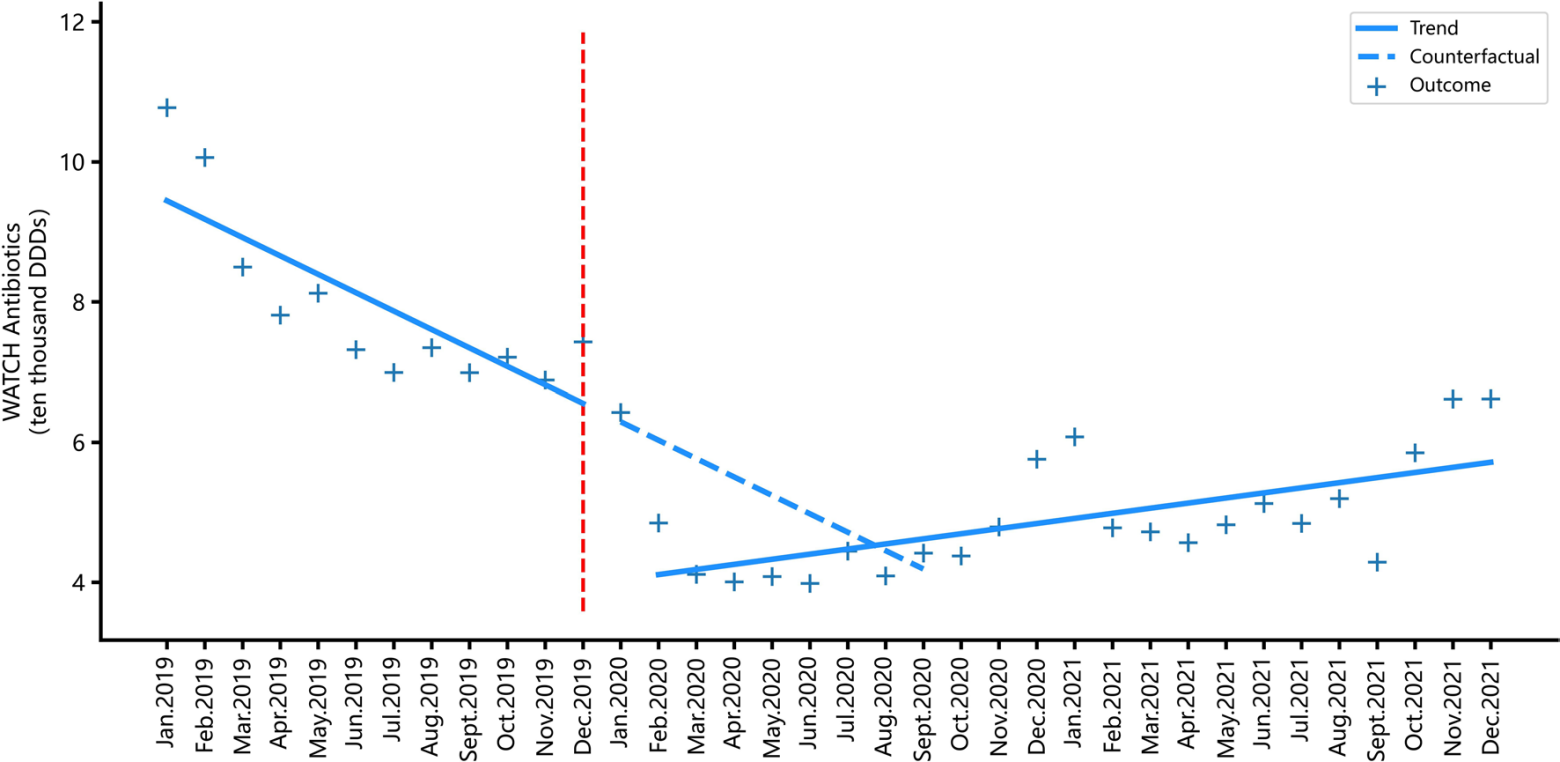
Monthly consumption trend of antibiotics from the Watch category prescribed in PHSs

As shown in Table 2, after the outbreak of the COVID-19 pandemic, the consumption of J01 antibiotics (antibacterials for systemic use) in PHSs showed a significant immediate decline immediately (coef. = − 2.712, *p* = 0.045), followed by a low-level increase with significance in the long-term (coef. = 0.205, *p* = 0.005). At the same time, the immediate impact of the outbreak of the COVID-19 pandemic on the consumption of J01C antibiotics (beta-lactam antibacterials, penicillins) had no statistical significance in the short term However, its consumption had a low-level increase in the long-term (coef. = − 0.117, *p* = 0.031). Similarly, the consumption of J01D antibiotics (cephalosporins, other beta-lactam antibacterial), J01M antibiotics (quinolone antibacterial) and J01F antibiotics (macrolides, lincosamides and streptogramins) all showed significant immediate declines in the short term after the COVID-19 pandemic outbreak (coef. = − 0.405, *p* = 0.020; coef. = − 0.083, *p* = 0.026; coef. = − 0.740, *p* < 0.001). It is particularly worth noting that there was no statistical significance in the immediate impact of COVID-19 on Access category antibiotic consumption, but a significant and rapid increase was observed in the long-term (coef. = 0.136, *p* = 0.018).On the contrary, the pandemic caused a significant immediate decrease of the consumption of Watch category antibiotics in the immediate (coef. = − 1.222, *p* < 0.001) and a slight increase in the long-term (coef. = 0.073, *p* < 0.001), with significant statistical differences (Figs. 3, 4, and Table 3).

**Table 3:** The segmented regression time series analysis of the antibiotic consumption in PHSs

Outcome indicators	Immediate effect				Long-term effect			
	Coef.	95% CI		*P*	Coef.	95% CI		*P*
J01 Antibacterials for systemic use (ten thousand DDDs)	− 2.712	− 46.327	50.671	0.045*	0.205	− 3.150	2.740	0.005**
J01C Beta-lactam antibacterials, Penicillins (ten thousand DDDs)	− 0.850	− 31.172	32.872	0.918	0.117	− 1.724	1.489	0.031*
J01D Cephalosporins, Other Beta-lactam antibacterials (ten thousand DDDs)	− 0.405	− 12.792	13.601	0.020*	0.025	− 0.912	0.863	0.133
J01F Macrolides, lincosamides and streptogramins (ten thousand DDDs)	− 0.740	− 1.416	2.897	< 0.001**	0.012	− 0.516	0.491	< 0.001**
J01G Aminoglycoside antibacterials (ten thousand DDDs)	− 0.114	− 0.705	0.932	0.005**	− 0.002	− 0.104	0.109	0.000**
J01M Quinolone antibacterials (ten thousand DDDs)	− 0.083	− 8.613	8.778	0.026*	0.033	− 0.719	0.653	0.844
J01X Other antibiotics in J01 (ten thousand DDDs)	− 0.001	− 3.550	3.552	0.632	0.023	− 0.965	0.918	0.927
Access category antibiotics (ten thousand DDDs)	− 0.970	− 29.687	31.627	0.931	0.136	− 1.976	1.704	0.018*
Watch category antibiotics (ten thousand DDDs)	− 1.222	− 18.900	21.345	< 0.001**	0.073	− 1.317	1.171	< 0.001**

*DDDs* defined daily doses, *Coef.* non-standardized coefficient, *CI* confidence interval

^*^p < 0.05; **p < 0.01

## Discussion

Our study assessed changes in antibiotic use during the COVID-19 pandemic. The segmented regression model revealed that antibiotic consumption declined significantly and the use pattern has changed before and after the COVID-19 pandemic outbreak.

In this study, a significant decline was observed in the overall antibiotic consumption in PHSs in 2020 and 2021 compared to the corresponding period in 2019, respectively. Interestingly, the interrupted time series analysis showed a significant immediate decline in overall antibiotic consumption, but a slow increase in the long-term. It is worth noting that antibiotics were partially unavailable in PHSs in China early in the pandemic. Physicians prescribed these drugs carefully, fearing delays in identifying potential COVID-19 patients due to insufficient diagnostic capabilities. The increasing antibiotic consumption after the effective control of the COVID-19 pandemic was potentially associated with the gradual restoration of medical order. According to previous studies in the UK [34] and Portugal [16], the number of antibiotic prescriptions and antibiotic consumption decreased in PHSs after the outbreak of the COVID-19 pandemic. However, the research report in Portugal [16] showed that the slight upward trend of the overall antibiotic consumption in the long-term was not statistically significant, which differs from the results of our study. We found that the overall antibiotic consumption had a statistically significant upward trend over the long-term. This result may be caused by the prevention and control policies on the COVID-19 pandemic in China. In the early stage of the COVID-19 pandemic, China issued many strict isolation and protection measures, such as requiring “Isolation at home,” and promoting “Do not go to hospitals unless indeed necessary” [17]. These measures reduced the number of outpatient visits and the incidence of influenza to some extent, causing antibiotics consumed in PHSs to drop sharply [17, 18]. However, after China successfully contained the COVID-19 pandemic rapidly and began to adopt normalized pandemic prevention and measures, antibiotic consumption began to rise again. This was possibly due to the gradual removal of a series of pandemic containing measures.

Moreover, after the COVID-19 pandemic outbreak, the consumption of Access category antibiotics in PHSs increased significantly in the long-term. However, the consumption of Watch category antibiotics decreased immediately but increased slightly in the long-term. Similarly, a previous study also demonstrated that the consumption of Watch category antibiotics declined after the COVID-19 pandemic outbreak [16]; and another study suggested that the proportion of the consumption of Access category antibiotics used in PHSs rise significantly during the COVID-19 pandemic [17]. This may be related to the strict restrictions and increased hygiene measures adopted during the COVID-19 pandemic, as well as the reduction of medical service contact and irrational prescriptions of antibiotics. Therefore, the COVID-19 pandemic may have optimized the use pattern of antibiotics and sent a positive signal for the management of antibiotic resistance in China [17, 32]. By assessing the changes in the consumption of three classes of antibiotics, i.e., J01D antibiotics (cephalosporins, other beta-lactam antibacterials), J01F antibiotics (macrolides, lincosamides and streptogramins) and J01M antibiotics (quinolone antibacterials), we found a significant immediate decrease of the consumption of all antibiotic classes in the short-term, which contributed to reducing antibiotic resistance [35, 36]. According to a study conducted in South Korea [37], the consumption of penicillin, cephalosporins and fluoroquinolones decreased during the COVID-19 pandemic, decreased by 54%, 32%, and 15%, respectively, compared to those in the previous year. This may be mainly attributed to the declined infectious diseases incidence caused by non-pharmaceutical interventions (i.e., hand-washing, face masks, physical distancing and travel restrictions), as well as the decline in medical services of PHSs due to the COVID-19 pandemic. However, it is worth noting that antibiotic use rebounded from the low level and increased slightly in the long term, indicating that the decline of antibiotic use during the pandemic may not last for a long time. The COVID-19 pandemic may have some positive impact on improving antibiotic misuse in PHSs [38]. At the same time, considering the fact that the majority of antibiotics are prescribed in PHSs in China [6–8], the declined antibiotic consumption and improved use patterns due to the COVID-19 pandemic bring a new opportunity for future policymakers to improve their antibiotic stewardship.

### Advantages and limitations

As for the strengths of this study, first we adopted a strong quasi experimental design, based on interrupted time series analysis, to assess the immediate and long-term impacts of the COVID-19 pandemic on antibiotic use by ATC classification and AWaRe category. When making antibiotic data analysis, we considered the seasonal factors and made corresponding adjustment. In addition, we completed an autocorrelation test to ensure the robustness of the results. This study provided the latest evidence for the changes in antibiotic use patterns in PHSs in Shandong Province of China.

However, there are several limitations in this study. First, due to the limited time and funds, we only obtained a small sample size for this study, and only collected the monthly antibiotic consumption data of 38 township hospitals. Second, as the data on patients and prescriptions were not available, we did not analyze the specific influencing factors causing the changes in antibiotic use patterns. Third, as the COVID-19 pandemic had a complex impact on the antibiotic consumption in each specific region or setting, the findings may not be extrapolated to other settings or regions. Therefore, it is necessary to carry out more researches in different settings.

## Conclusion

To sum up, our study suggests that the COVID-19 pandemic has an in-depth impact on antibiotic consumption in PHSs in Shandong, China. After the outbreak of COVID-19 pandemic, the overall antibiotic consumption declined significantly in the short-term. However, it showed a increase over the long-term. Consistent priority use of penicillin and cephalosporin should be of great concern in the PHSs in Shandong, China. In addition, the COVID-19 pandemic caused a rapid increase in the consumption of Access category antibiotics in the long-term but an immediate significant decrease in the consumption of Watch category antibiotics. This may have improved the antibiotic use patterns in PHSs to some extent. Therefore, the COVID-19 pandemic may bring an opportunity for better management of antibiotics use in China. In future studies, it is necessary to monitor antibiotic consumption and collect data on patients using antibiotics and related prescriptions s to better explore the impact of the COVID-19 pandemic on antibiotic use. In this way, we may find the direct factors causing the aforementioned changes. Policy-makers should seize the opportunity to overcome the challenges faced by antibiotic use under the COVID-19 pandemic in PHSs in China to improve their management to a higher level.

## Supplementary Information

**Additional file 1. Table S1**. List of the antibiotics analyzed in this study.

## Data Availability

The datasets used and analyzed during the current study are available from the corresponding author upon reasonable request.

## Abbreviations

PHSs: Primary healthcare settings
COVID-19: Coronavirus Disease-19
DDDs: Defined daily doses
AWaRe: Access, Watch, Reserve
AMR: Antimicrobial resistance
WHO: World Health Organization
ATC: Anatomical Therapeutic and Chemical
GDP: Gross Domestic Product
DDD: Defined daily dose
DPP: DDD equivalence per package
J01: Antibacterials for systemic use
J01C: Beta-lactam antibacterials, penicillins
J01D: Cephalosporins, other beta-lactam antibacterials
J01F: Macrolides, lincosamides and streptogramins
J01G: Aminoglycoside antibacterials
J01M: Quinolone antibacterials
J01X: Other antibiotics in J01
Coef.: Non-standardized coefficient
CI: Confidence interval
