# Supplementary material for: Antibiotic use in township hospitals during the COVID-19 pandemic in Shandong, China
Source: Antimicrob Resist Infect Control. 2022 Dec 24;11:164. doi: 10.1186/s13756-022-01206-8 (PMC9789504; doi:10.1186/s13756-022-01206-8)
Supplement: Supplementary file 1 — Additional file 1. Table S1. List of the antibiotics analyzed in this study. [file 13756_2022_1206_MOESM1_ESM.docx]

**Table S1.** List of the antibiotics analyzed in this study

| **Category*^a^*** | **Antibiotic**  **(generic name)** | **Antibiotic (classification)** | **ATC-5 Code*^b^*** | **ATC-4 class*^b^*** | **ATC-3 group*^b^*** |
| --- | --- | --- | --- | --- | --- |
| Access | Amoxicillin | Penicillins | J01CA04 | J01CA | J01C |
|  | Benzylpenicillin | Penicillins | J01CE01 | J01CE | J01C |
|  | Cefradine | First-generation-cephalosporins | J01DB09 | J01DB | J01D |
|  | Clindamycin | Lincosamides | J01FF01 | J01FF | J01F |
|  | Metronidazole_IV | Imidazoles | J01XD01 | J01XD | J01X |
|  | Tinidazole_IV | Imidazoles | J01XD02 | J01XD | J01X |
|  | Gentamicin | Aminoglycosides | J01GB03 | J01GB | J01G |
|  | Ampicillin | Penicillins | J01CA01 | J01CA | J01C |
|  | Amoxicillin/clavulanic-acid | Beta-lactam/beta-lactamase-inhibitor | J01CR02 | J01CR | J01C |
|  | Oxacillin | Penicillins | J01CF04 | J01CF | J01C |
|  | Benzathine-benzylpenicillin | Penicillins | J01CE08 | J01CE | J01C |
|  | Amikacin | Aminoglycosides | J01GB06 | J01GB | J01G |
|  | Ampicillin/sulbactam | Beta-lactam/beta-lactamase-inhibitor | J01CR01 | J01CR | J01C |
|  | Ornidazole_IV | Imidazoles | J01XD03 | J01XD | J01X |
|  | Nitrofurantoin | Nitrofuran-derivatives | J01XE01 | J01XE | J01X |
|  | Cefazolin | First-generation-cephalosporins | J01DB04 | J01DB | J01D |
|  | Cefazolin | First-generation-cephalosporins | J01DB04 | J01DB | J01D |
|  | Cefalexin | First-generation-cephalosporins | J01DB01 | J01DB | J01D |
|  | Amoxicillin/clavulanic-acid | Beta-lactam/beta-lactamase-inhibitor | J01CR02 | J01CR | J01C |
|  | Cloxacillin | Penicillins | J01CF02 | J01CF | J01C |
|  | Cefadroxil | First-generation-cephalosporins | J01DB05 | J01DB | J01D |
|  | Ceftriaxone | Third-generation-cephalosporins | J01DD04 | J01DD | J01D |
| Watch | Cefuroxime | Second-generation-cephalosporins | J01DC02 | J01DC | J01D |
|  | Levofloxacin | Fluoroquinolones | J01MA12 | J01MA | J01M |
|  | Azithromycin | Macrolides | J01FA10 | J01FA | J01F |
|  | Erythromycin | Macrolides | J01FA01 | J01FA | J01F |
|  | Ceftazidime | Third-generation-cephalosporins | J01DD02 | J01DD | J01D |
|  | Cefaclor | Second-generation-cephalosporins | J01DC04 | J01DC | J01D |
|  | Piperacillin/tazobactam | Beta-lactam/beta-lactamase-inhibitor anti-pseudomonal | J01CR05 | J01CR | J01C |
|  | Piperacillin | Penicillins | J01CA12 | J01CA | J01C |
|  | Norfloxacin | Fluoroquinolones | J01MA06 | J01MA | J01M |
|  | Roxithromycin | Macrolides | J01FA06 | J01FA | J01F |
|  | Cefixime | Third-generation-cephalosporins | J01DD08 | J01DD | J01D |
|  | Clarithromycin | Macrolides | J01FA09 | J01FA | J01F |
|  | Cefotaxime | Third-generation-cephalosporins | J01DD01 | J01DD | J01D |
|  | Lincomycin | Lincosamides | J01FF02 | J01FF | J01F |
|  | Ciprofloxacin | Fluoroquinolones | J01MA02 | J01MA | J01M |
|  | Azlocillin | Penicillins | J01CA09 | J01CA | J01C |
|  | Cefditoren-pivoxil | Third-generation-cephalosporins | J01DD16 | J01DD | J01D |
|  | Moxifloxacin | Fluoroquinolones | J01MA14 | J01MA | J01M |
|  | Meropenem | Carbapenems | J01DH02 | J01DH | J01D |
|  | Imipenem/cilastatin | Carbapenems | J01DH51 | J01DH | J01D |
|  | Vancomycin_IV | Glycopeptides | J01XA01 | J01XA | J01X |
|  | Ceftizoxime | Third-generation-cephalosporins | J01DD07 | J01DD | J01D |
|  | Mezlocillin | Penicillins | J01CA10 | J01CA | J01C |
|  | Cefotiam | Second-generation-cephalosporins | J01DC07 | J01DC | J01D |
|  | Cefotiam | Second-generation-cephalosporins | J01DC07 | J01DC | J01D |
|  | Levofloxacin | Fluoroquinolones | J01MA12 | J01MA | J01M |
|  | Cefminox | Second-generation-cephalosporins | J01DC12 | J01DC | J01D |
|  | Spiramycin | Macrolides | J01FA02 | J01FA | J01F |
|  | Cefoxitin | Second-generation-cephalosporins | J01DC01 | J01DC | J01D |
|  | Dirithromycin | Macrolides | J01FA13 | J01FA | J01F |
|  | Pipemidic-acid | Quinolones | J01MB04 | J01MB | J01M |
| Reserve | Fosfomycin_IV | Phosphonics | J01XX01 | J01XX | J01X |

*a* Antibiotics were classified according to the WHO AWaRe categories (version 2021); *b* Antibiotics were coded in accordance with the Anatomical Therapeutic Chemical (ATC) classification system.
